# Protein Large Language Models Can Predict *Flavivirus* Protease Target Specificity

**DOI:** 10.1021/acsomega.5c13455

**Published:** 2026-04-01

**Authors:** Rafael Montilla, Letícia Dias Lima Jedlicka, Uilla Barcick, Murilo Salardani, Alison Felipe Alencar Chaves, Gloria Gallo, Marcela Guimarães, Camila Coelho, Larissa Slivka, André Zelanis, Martin Würtele

**Affiliations:** † Department of Science and Technology, Biochemistry and Structural Biology Laboratory, 28105 Federal University of São Paulo - UNIFESP, São José dos Campos, São Paulo 12231-280, Brazil; ‡ Department of Science and Technology, Functional Proteomics Laboratory, Federal University of São Paulo - UNIFESP, São José dos Campos, São Paulo 12231-280, Brazil; § Institute of Health and Biological Studies, Federal University of Southern and Southeastern Pará - UNIFESSPA, Marabá, Pará 68507-590, Brazil; ∥ Laboratory of Applied Toxinology, Center of Toxins, Immune-Response and Cell Signaling (CeTICS), Instituto Butantan, São Paulo 05503-000, Brazil

## Abstract

Viral proteases are essential enzymes in many viral strains, playing a crucial role in the viral replication cycle. They are key targets for antiviral drug development and have significant implications for viral pathogenesis. To address the issue of *Flavivirus* protease substrate promiscuity, Yellow Fever virus protease (YFP), West Nile Virus Protease (WNP), Zika virus protease (ZVP), Usutu Virus Protease (UVP), and Rocio Virus Protease (RVP) were recombinantly expressed in *E. coli* BL21­(DE3) and purified. Mass spectrometric Proteomic Identification of protease Cleavage Sites (PICSs) was performed using peptide libraries derived from a murine cell line lysate. A surprisingly high promiscuity in protease substrate specificity was detected for all five viral proteases, with a recurrence of arginine in the P1 position. Using homology modeling, specific subsites could be identified. However, the promiscuity of peptide binding was difficult to elucidate using these models. For these reasons, the ProtTrans protein language model (pLM) was used and fine-tuned with the obtained peptide sequences. The ProtTrans T5-Encoder model, originally trained to predict same protein-chain amino acids using a huge size of protein sequence data, when fine-tuned with target peptides from the PICS experiments and decoy peptides, could classify each of these groups with up to 76% test-set accuracy. Dimensionality reduction indicated that the T5 embeddings could indeed contain similar information, which was useful for recognizing protein–peptide interactions. These results confirm the usefulness of pLMs for the prediction of protein–protein interactions and thus have important implications for antiviral drug design.

## Graphical abstract

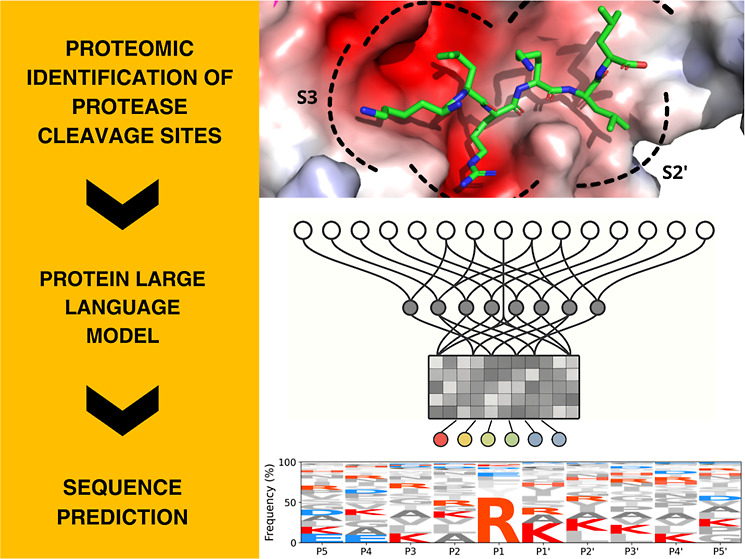

## Introduction

The recent emergence and reemergence of human viruses represents a serious health threat.^1–4^ Viruses in the Flaviviridae family and the *Flavivirus*/*Orthoflavivirus* genus are no exception, with several outbreaks of these enveloped, positive-stranded RNA viruses recently reported. Zika virus is transmitted by infected *Aedes* mosquitoes. Infection caused by this virus has been associated with congenital brain abnormalities, including microcephaly, as well as the development of Guillain–Barré syndrome.^5,6^ Currently, there is no approved vaccine or specific antiviral treatment available for Zika virus disease.^7–9^ The yellow fever virus is transmitted by the same mosquito.^10^ West Nile virus is transmitted by *Culex* sp. mosquitoes.^11^ It is neuropathogenic in humans, horses, and birds. Its symptoms present as a febrile disease, associated with changes in the level of consciousness and intense muscle weakness, later characterized as a human meningoencephalitis.^12^ They were first described in America in 1999, in the United States.^12^ The Usutu virus (USUV) is also an arbovirus and is a member of the antigenic complex of the Japanese encephalitis virus.^13^ The first case of USUV infection in humans was reported in the early 1980s in Africa, with later cases reported in Europe.^14^ The symptoms caused by the Usutu virus vary, ranging from fever and headache in milder cases, to neurological disorders in more severe cases.^15^ The Rocio virus (ROCV) was responsible for an outbreak of encephalitis in Brazil in the 1970s. Its symptoms range from fever and headache in milder cases, to neurological disorders in more severe instances of the infection.^16^

The viruses of the genus *Flavivirus* contain a single-stranded RNA genome with a positive strand. This genome is initially translated into a single polyprotein chain in infected host cells. The resulting precursor polyprotein is composed of three structural proteins, usually proteins C (capsid), protein M (membrane), protein E (envelope), and several nonstructural proteins, such as NS1, NS2A, NS2B, NS3, NS4, NS5.^17–19^ Bioinformatic analyses and comparisons with other proteins from related viruses reveal that NS2B proteins and part of NS3 form a serine protease that processes the precursor polyprotein. The C-terminal part of the NS3 protein forms a helicase. Also, at the C-terminus of the precursor polyprotein (NS5 region), there is a protein with methyltransferase activity and a protein with RNA-polymerase activity. The nonstructural proteins synthesized by the virus are essential for RNA replication, viral assembly, evasion of the host’s immune system, and its pathogenesis.^20,21^

Viral proteases, together with polymerases, are well studied and promising targets for antiviral drug design. They are thought to have a rather specific substrate specificity. However, recent results indicate that—at least *in vitro*—proteases like Mpro from SARS-CoV-2 have a far more promiscuous range of substrate specificity than thought.^22,23^ For these reasons, we have cloned and recombinantly expressed and purified Yellow Fever virus protease (YFP),^10^ West Nile Virus Protease (WNP),^12^ Zika virus protease (ZVP),^5^ Usutu Virus Protease (UVP),^13^ and Rocio Virus Protease (RVP)^16,24^ in *E. coli* BL21­(DE3). After biochemical characterization of the activity of the obtained recombinant proteases, Proteomics Identification of protease Cleavage Sites (PICS) was carried out to identify protease subsite specificities using murine cell line peptide libraries. The obtained results showed an unexpected promiscuity in subsite specificity for all five tested flaviviral proteases. To analyze this phenomenon, bioinformatics and homology modeling analyses were carried out as well as LLM analysis using the ProtTrans transformer neural network model.

Recent developments indicate that large language models (LLMs) can be used to predict protein–protein interactions.^25–27^ LLMs are deep learning algorithms that can perform natural language processing (NLP) tasks after being pretrained on vast amounts of data. They are generally based on the transformer architecture, which uses the so-called *attention mechanism* to try to determine the relative importance of tokens in a sequence to other tokens in that same sequence.^28^ Because of similarities between human language coding and protein sequences, LLMs have been recently adapted for prediction of protein sequence-related properties, like secondary structure and protein targeting.^29,30^ Here, we address the question of whether LLMs can be used to predict flaviviral protease substrate promiscuity and specificity. For this end, we have used the pretrained ProtTrans protein language model (pLM)^31^ and fine-tuned it with data obtained from mass-spectrometry data obtained after proteomic identification of protease cleavage sites experimentally using five different recombinant flaviviral proteases.

Basically, ProtTrans and related pLM models have been trained by exposing LLM architectures to large amounts of the known proteome data of all sequenced living organisms. The presumed rationale behind this approach is that by self-supervised training using the attention mechanism and huge amount of protein sequences, the LLM will create approximated descriptions of hidden protein-coded information, which would be reminiscent of deciphering a higher order protein “life code”. Using this postulated LLM coded “reminiscent memory” can thus presumably be helpful in analyzing proteins for extracting hidden information. After fine-tuning the model with additional information, the LLMs can, as has been demonstrated, indeed predict important structural and functional properties of proteins, e.g., secondary structure and their most probable compartmentalization in the cell.^31^

## Methods

### Protein Expression and Purification

The five proteases, Yellow Fever Protease (GenBank entry ON323052.1), Zika Virus Protease (GenBank entry KU729217.2), West Nile Protease (GenBank entry HQ671722.1), Usutu Virus Protease (GenBank entry MT241508.1), and Rocio Virus Protease (GenBank entry MF461639.1), were synthesized (GenScript, USA) and cloned into the pET21a plasmid to create expression constructs with N-terminal His-tags for recombinant expression in *E. coli* BL21­(DE3), purification by affinity chromatography and size exclusion chromatography, as previously described.^9^

### Proteomic Identification of Protease Cleavage Sites (PICS)

Protease subsite specificity was carried out using the PICS protocol.^32^ Peptide libraries were prepared as previously described.^32^ Briefly, libraries were generated by digestion of lysates of murine melanoma cells (B16F10 cell line, CRL 6475, American Type Culture Collection, USA) with endoproteinase Glu-C (Sigma, USA). Free sulfhydryls were protected with iodoacetamide, and primary amines (N-terminal α-amines and lysine ε-amines) were protected by reductive dimethylation. Peptide libraries were purified by using solid-phase cartridges (SepPak, C18, Waters, USA) and stored at −80 °C until use. Each viral protease (2 μg) was incubated for 18 h at 30 C with 200 μg of the peptide library (1:100, protease:library ratio), in 50 mM HEPES buffer pH 8.5, containing 5 mM CaCl_2_ (final concentration). After incubation, 5 μL of 20 mM sulfo–NHS–SS-biotin, prepared in DMSO (Thermo Fisher, USA), was added to each reaction mixture, and the mixtures were left at room temperature for 2 h. Biotinylated cleavage products were incubated at 4 °C for 16 h with 1.2 mL of streptavidin-sepharose slurry (Cytiva, USA) in 50 mM HEPES, 150 mM NaCl, pH 7.5. After the addition of the elution buffer (40 mM DTT in 50 mM HEPES, 150 mM NaCl, pH 7.5), incubation was prolonged for 90 min at room temperature.

### LC–MS/MS and Proteomics Data Analysis

Prime-side cleavage product peptides derived from the incubation with viral proteases were desalted with Sep-Pak Vac C18 1 cc (Waters, USA), vacuum-dried, and resuspended in 10 μL of 0.1% formic acid. The peptide mixture was injected into a Vanquish Neo/Orbitrap Exploris 480 system equipped with a FAIMS source and a trap-and-elute column system. The mass spectra were acquired in Data-Dependent Acquisition mode with a 90 min linear gradient of acetonitrile in 0.1% formic acid. The raw files were analyzed through the Trans Proteomic Pipeline platform^33^ using the Comet search engine^34^ against the SwissProt database restricted to the taxa Rodentia (February 2024 UniProt release with 28,012 entries) with a decoy database appended in which all entries had their sequences reverse-oriented. Mass tolerance was set to 10 ppm for both parent and fragment ions. Semispecific enzyme specificity was selected (i.e., semi-Glu-C), with up to 2 missed cleavages. Thioacylation of peptide N-terminus (+87.998285 Da), iodoacetamide derivative of cysteine (+57.021464 Da), and dimethylation of the lysine ε-amino group (+28.031300 Da) were selected as static modifications, whereas oxidation of methionine (+15.994919 Da) was selected as variable modification. Search results were filtered with PeptideProphet probability set to ≥99%, corresponding to a False Discovery Rate (FDR) of <1%.

### Homology Modeling, Supervised Fine-Tuning, and Classification Using a Protein Large Language Model

Viral protease models with binding peptides were constructed using the MODELLER homology modeling software^35^ and a WNP complex with bovine pancreatic trypsin inhibitor (BPTI) structure as a template (PDB ID 2IJO). Images were rendered using PyMOL^36,37^ using the APBS electrostatics plugin.^38^ The ProtT5-XL UniRef50 encoder model (pretrained with the UniRef50 data set) was finetuned using the LoRA (Low Rank Adaption of LLMs) framework as described in ProtTrans repository (https://github.com/agemagician/ProtTrans).^39^ To rigorously evaluate the model’s performance, two distinct training strategies were employed: A single-protease strategy, in which five independent models were trained using peptide data specific to each viral protease (YFP, WNP, ZVP, UVP, or RVP) to assess virus-specific constraints, and an all-protease (pooled) peptide strategy, in which all unique cleavage sites were combined into a unified data set. For data set construction across both strategies, positive experimental samples were matched in a 1:1 ratio with negative decoy samples generated by in silico processing of the mouse proteome (UniProt ID UP000000589). These decoys consisted of random fragments absent from the positive set and were balanced to mirror the proportion of canonical (P1 = Arg) versus noncanonical peptides found in the experimental data, ensuring the model learned subtle sequence features rather than simple motif counting. The final data set consisted of 2918 unique peptides, comprised of 1476 positive targets (experimentally validated cleavage sites) and 1442 negative decoys. Validation was performed using a rigorous 80/20 split with 20% of the data held out as an independent test set for accuracy assessment. tSNE and ROC-AUC analysis were carried out using scikit-learn.^40^

## Results

In order to obtain more data about flaviviral protease specificity, in this work, Yellow Fever virus protease (YFP), West Nile Virus Protease (WNP), Zika virus protease (ZVP), Usutu Virus Protease (UVP), and Rocio Virus Protease (RVP) were produced recombinantly in *E. coli* BL21­(DE3). All five serine proteases were initially positively tested for activity using a general flaviviral protease-substrate FRET-peptide (Bz-Nle-KRR-AMC; data not shown). Next, the proteases were incubated in vitro with a mouse Glu-C peptide library, specially prepared for PICS analysis, which allowed for the identification of the peptides processed by each protease.

For each viral protease, an average of 658 unique peptides (cleavage sites) could be detected (Data sets S1–S5). Classically, the “canonical” flaviviral substrate motif is described as a dibasic sequence at the P2–P1 positions (typically Lys–Arg or Arg–Arg) often followed by a small polar residue at P1′.^41–43^ However, our PICS analysis revealed broad subsite specificities. Using the presence of the highly conserved Arginine at P1 as the strict definition of a canonical binder, approximately 40% of the identified peptides was classified as “canonical”, with significant variability also observed at the P2 position. All obtained peptides for each virus are published in the data sets described in the Supporting Information.

Figure 1 shows the relative amino acid frequency for each subsite position for all five viral proteases. The most common amino acids at each subsite (P5 to P5′) position in the substrates of all proteases are described in Table S1. As expected, the most common amino acid found at the P1 position was Arginine. The frequency of finding this residue in this position ranges from 35% in UVP to 60% in WNP. Additional amino acids with a higher-than-average representation are Lysine in P1′ to P4′ (in some cases like in YFP, reaching almost 30% of frequency in P1′) and Leucine in different positions. Interestingly, all of the other amino acids showed less pronounced distributions on the different peptide positions. There was, however, as shown in Figure 1, a slight preference for certain amino acids in the different peptide positions.

**Figure 1:**
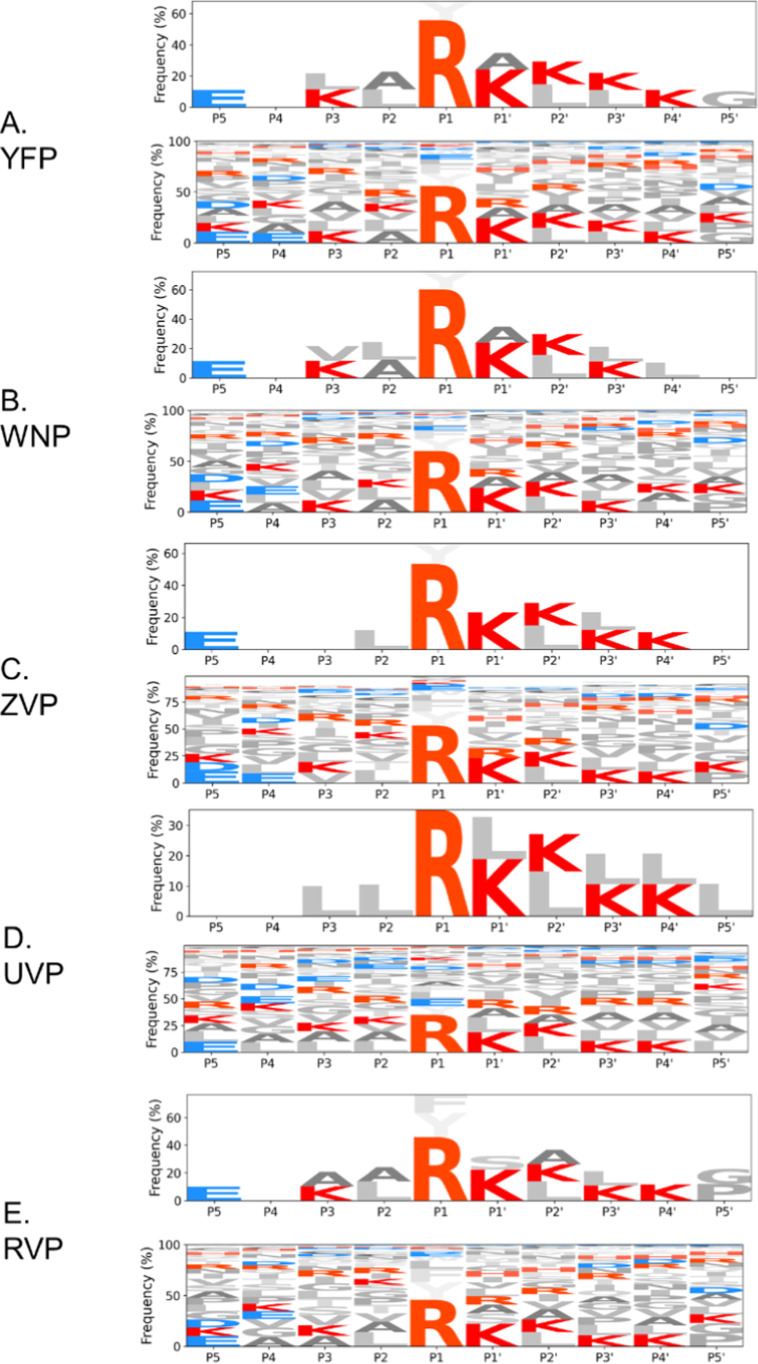
Relative frequencies of amino acids from the PICS assay, showing high promiscuity of target peptides. Upper images include amino acids with frequencies above 10%. Lower images include all amino acids. (a) YFP: Yellow Fever virus protease. (b) WNP: West Nile Virus Protease. (c) ZVP: Zika virus protease. (d) UVP: Usutu Virus Protease. (e) RVP: Rocio Virus Protease.

Overall, these surprising results indicate a high degree of promiscuity of the tested flaviviral proteases. Because of the large number of identified peptides, we next analyzed whether the single proteases showed a specific pattern of cleavage. Contrary to our expectations, however, we found a large number of conserved peptides that were cleaved by several of the tested proteases. As shown in Table 1, the percentage of common peptides varied between 37.3% and 76.5%, indicating that indeed flaviviral proteases have a functionally conserved target peptide specificity, possibly due to their conserved structure (amino acid identities between 48.5% and 71.4%).

**Table 1:** Percentage of Common Target Peptides between the Proteases Identified by the PICS Assay^a^

	YFP	WNP	ZVP	UVP	RVP
YFP	100	76.5	68.2	37.3	65.3
WNP		100	70.1	38.6	64.8
ZVP			100	39.6	68.3
UVP				100	39.9
RVP					100

a YFP: Yellow Fever virus protease. WNP: West Nile Virus Protease. ZVP: Zika virus protease. UVP: Usutu Virus Protease. RVP: Rocio Virus Protease.

To define the biochemical boundaries of this broad substrate tolerance, we compared the consensus motifs of canonical (P1 = Arg) versus noncanonical binders (Figure S1). Detailed sequence analysis revealed a distinct hydrophobic compensatory mechanism. Noncanonical substrates exhibit significantly higher general hydrophobicity (mean score −2.1 on the Kyte–Doolittle scale) compared to canonical binders (−6.6). In the absence of the P1-Arginine, the S1 subsite preferentially selects for bulky hydrophobic residues (specifically Tyrosine, Phenylalanine, and Leucine). Furthermore, this compensation extends to the prime side, where the P1′ position displays a marked enrichment for Leucine and Lysine, suggesting that stabilizing hydrophobic interactions and charge conservation at the S1–S1′ interface are critical when the canonical motif is absent.

To determine binding specificity of the proteases and whether this hydrophobic compensatory mechanism is uniformly utilized by all five proteases or represents a strain-specific feature, we performed Principal Component Analysis (PCA) and discriminant feature profiling on the individual viral data sets (Figure S2). This analysis revealed that while the vast majority of substrates are compatible with all five proteases, the viruses display distinct functional profiles. West Nile Virus (WNV) behaves as the strictest canonical specialist, exhibiting the highest frequency of the classical P1-Arginine motif (∼60%). In contrast, Usutu Virus (UVP) displays the most divergent specificity profile with the lowest adherence to the P1-Arginine rule (∼35%), while Rocio Virus (RVP) drives the hydrophobic signal through a unique preference for residues such as Tyrosine and Phenylalanine at P1. Thus, while the structural capacity for promiscuity is conserved across the genus, diverse binding specificities can be detected in the different strains.

To interpret the PICS data from a structural perspective, we performed simple homology modeling to visualize the peptide–protease interface. We utilized the crystal structure of West Nile Virus protease in complex with BPTI (PDB code 2IJO) as the template, as it currently represents the closest available structural homologue of all five closely related flaviviral proteases bound to a peptide-like substrate mimic.^44^ While the overall sequence identity to the template ranges from 49.8% (YFP) to 71.4% (UVP), the sequence identity conservation within the substrate-binding cleft is significantly higher, ranging from 71.1% to 76.3% (when calculated using all protease residues within 6.0 Å of the peptide substrate). By mapping the identified consensus sequences into the active site of this template, we could assess potential steric and electrostatic complementarity between the protease subsites and the preferred amino acids at positions P3–P3′. The homology modeling was carried out using the MODELLER software.^35^ To validate these structural models, predictions were also generated using AlphaFold 3,^45^ which confirmed the overall binding geometry with root Mean Square Deviations (RMSDs) for the peptide Cα atoms ranging from 0.8 Å (in case of the WNP model) to 2.3 Å (in the case of the UVP model).

The electrostatically contoured molecular surface of the proteases, together with the substrate subsites and peptide homology models, is shown in Figure 2. From the structural point of view, the electrostatic surface representation indicates that most of the molecular surfaces of the proteases are negatively charged or nonpolar. This is in accordance with the finding that most peptides contained more positively charged Lysines and Arginines in their sequences (Figure 2), as well as the occurrence of several nonpolar amino acids. For modeling, a peptide for each protease was chosen that contained the most frequent amino acid in each of the 6 peptide substrate positions (P3–P3′). As BPTI binds and inhibits target proteases in a mode similar to the native peptide-binding mode, albeit in such a way that does not favor peptide hydrolysis, the side chains of the BPTI-WNP virus structure (PDB code 2IJO) could be used to identifying the substrate subsites.^44^ As expected, the S1′ and S3′ are in an adjacent overlapping position on the protease surface, indicating that the most common residue in these positions (Lysine) would not favor peptide binding and hydrolysis due to steric and charge proximity restraints, if present in both binding sites. Coincidently, we indeed found that while Lysine/Arginine is indeed frequent at P1′ (28%) and P3′ (17%) individually, their simultaneous occurrence is significantly depleted in the experimental data set (Observed: 2.6% vs Expected: 4.7%). The side chains of all other peptide positions seemed to bind in a structurally favorable way in the protease subsites.

**Figure 2:**
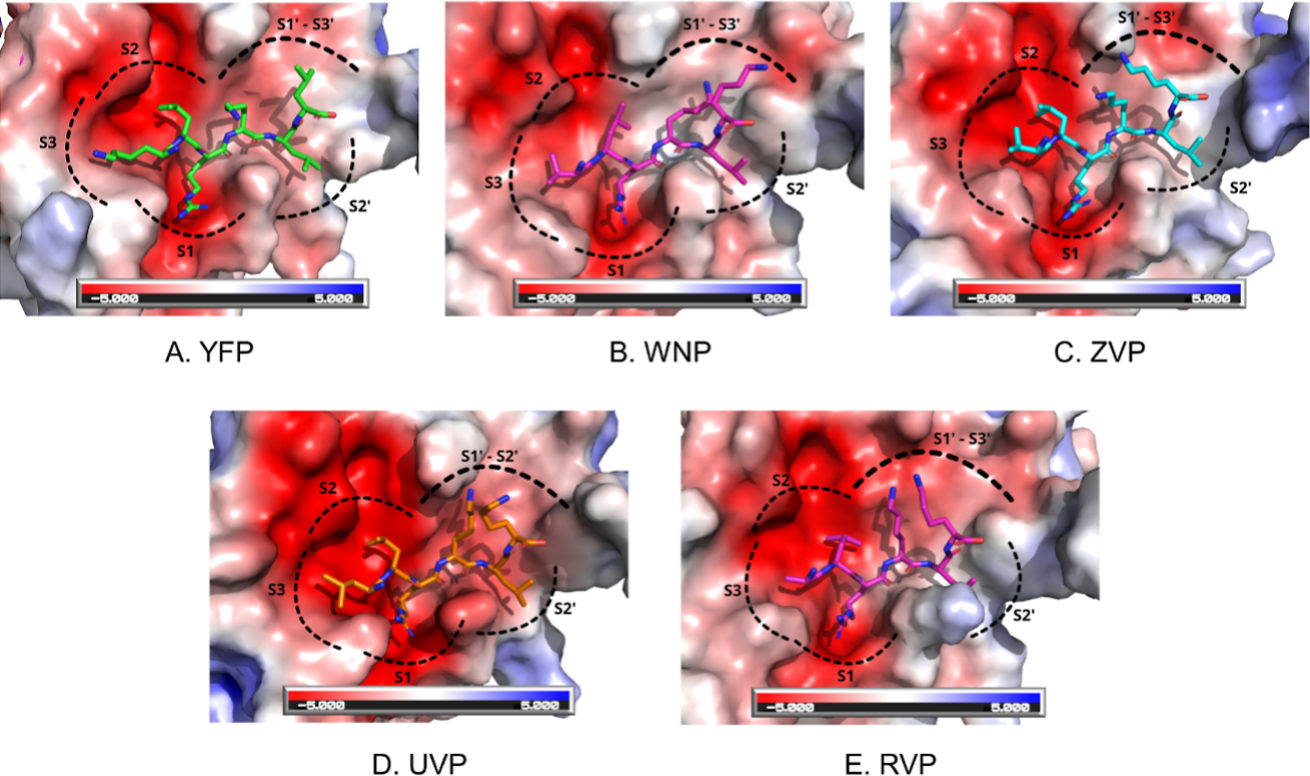
Homology modeling. The peptides identified in the PICS analysis for each viral protease were homology modeled with MODELLER using a BPTI-WNP virus structure (PDB code 2IJO) as a template. The flaviviral protease-binding site is shown as an electrostatic potential surface. (a) Yellow Fever virus protease (YFP) is modeled with a KLRKLL peptide. (b) West Nile Virus Protease (WNP) modeled with a KARKLK peptide. (c) Zika virus protease (ZVP) modeled with a VLRKLK peptide. (d) Usutu Virus Protease (UVP) modeled with a LLRKLK peptide. (e) Rocio Virus Protease (RVP) modeled with an ALRKLK peptide.

From a structural perspective, steric and electrostatic constraints suggest that while some peptides may exhibit stronger binding affinity, more than just the canonical peptides could bind and undergo hydrolysis by the tested flaviviral proteases, in accordance with the obtained PICS results. However, due to the large number of obtained target peptides, a more general approach to this modeling task had to be found. To this end, the ProtTrans neural network transformer model was tested. As the proteases showed a high degree of similarity in relation to their substrate specificity, the ProtTrans model was fine-tuned both with single and pooled peptides from all proteases. To obtain a classifier model, a set of decoy peptides randomly derived from the mouse proteome was used as a negative control. The training history of the LORA fine-tuned pooled all-protease model is shown in Figure 3. The obtained model (Data set S6) indicates significant improvement of the accuracy of training with the fine-tuning when compared to initial pretrained T5 encoder model. After fine-tuning of both the T5 encoder model and the classifier head, a training set accuracy of 81.3% and a test set accuracy of 76.3% could be obtained (Table 2).

**Figure 3:**
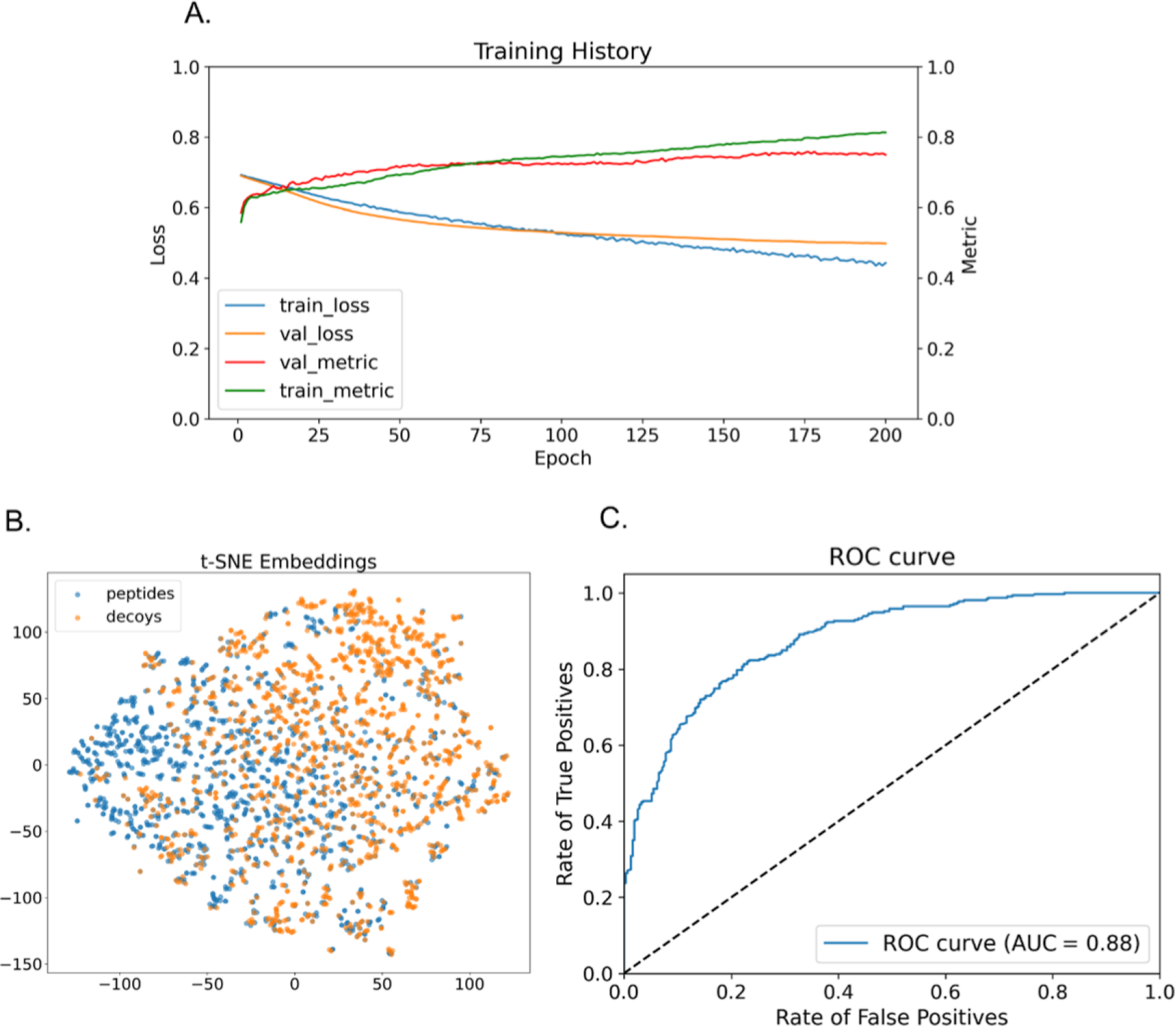
pLM finetuning using target and decoy peptides for all flavivirus proteases. (a) Training history curves for training and validation sets showing training loss function (train_loss) and validation loss function (val_loss) as well as the training accuracy (train_metric) and validation accuracy (val_metric). (b) t-SNE 2D projection representing partial separation of target peptides (blue) and decoy peptides (yellow) embeddings from the fine-tuned ProtTrans T5encoder model. (c) Receiver operating characteristic (ROC) curve as a false positive rate vs true positive rate plot of target peptides and decoy classification using the fine-tuned ProtTrans T5 encoder model with area under the curve (AUC) value calculated.

**Table 2:** pLM Finetuning with the ProtTrans T5 Encoder and Binary Classification Head^a^

train set all-protease model					test set all-protease model				
	total	classified as target	classified as decoy	accuracy [%]		total	classified as target	classified as decoy	accuracy [%]
canonical targets	462	337	125	72.9	canonical targets	124	89	35	71.8
noncanonical targets	715	604	111	84.5	noncanonical targets	175	136	39	77.7
total targets	1177	941	236	79.9	total targets	299	225	74	75.3
canonical decoys	579	79	500	86.4	canonical decoys	154	36	118	76.6
noncanonical decoys	578	133	445	77.0	noncanonical decoys	131	30	101	77.1
total decoys	1157	212	945	81.7	total decoys	285	66	219	76.8

	total	correctly classified	wrongly classified	accuracy [%]		total	correctly classified	wrongly classified	accuracy [%]
total peptides	2334	1886	448	80.8	total peptides	584	444	140	76.0

a Results of the trained model are given for the train set and test set, where the train set contained target peptides of all five flaviviral proteases, and the test set contained 20% of the data for model validation purposes.

To corroborate the results, the ProtTrans T5 encoder model was also trained with individual virus proteases separately. The resulting tables and training histories are shown in the Supporting Information (Tables S2–S6 and Figure S3). The obtained accuracies are very similar to those of the ProtTrans T5 encoder model trained with the complete target peptide database. Total test set accuracies range from 66.5% (UVP) to 75.3% (YFP). The average training accuracy of these models (Data sets S7–S11) was 81.7%, and the average test accuracy of these models was 71.9%.

We next analyzed whether the output embeddings of the ProtTrans T5 encoder model could be separated by clustering after 2D projection using t-Distributed Stochastic Neighbor Embedding (t-SNE). Since embeddings are multidimensional representations of a given sequence, this technique is a suitable option to address that question. Indeed, the t-SNE 2D projection of the embeddings of target peptides and decoy peptide formed distinct clusters, even though there was some overlap in both clusters, as shown in Figures 3B and S4.

Finally, for the model performance assessment, receiver operating characteristic (ROC) curves were calculated for the obtained binary classification model. The obtained ROC-AUC analysis shows results that are in line with the previously described analysis, with, e.g., a rate of 80% true positive classifications correlated to a 20% false positive classification and an AUC value of 0.88 (Figure 3C). Together, these results corroborate the hypothesis that protein LLM is able to distinguish sequences generated by high promiscuity proteases.

## Discussion

Viral proteases are thought to have a rather high rate of specificity for their target peptides. In the case of flaviviral proteases, they are, for example, known to often cut after an arginine residue in the P1 position. Recent results, however, have indicated that viral proteases do have some degree of promiscuity toward substrates.^22^ In order to gain more insight into the range of substrate specificity and promiscuity in flaviviral proteases, in this work, five recombinant flaviviral proteases were analyzed for substrate specificity using a proteomics approach. Interestingly, whereas an average of 39.7% of the identified peptides indeed displayed an arginine in the P1 position, a whole set of noncanonical peptides could be identified in this work. The results presented here are thus of importance for the evaluation of off-target effects, which have been described as important for the pathogenesis of several viruses. Of course, caution is needed when interpreting the obtained in vitro results using preprocessed peptide libraries and transferring them to in vivo predictions. This is not necessarily the case if the purpose is understanding flaviviral protease specificity, as well as for viral protease inhibitor design. In this sense, it is interesting to note that besides the canonical arginine in the P1 position, only a few elevated frequencies, e.g., of Lysines in P1′-P4′ and Leucine residues in several positions, could be detected, and no truly elevated sequence frequency of other specific amino acids could be found. Even in the case of P1 Arginine, only a frequency of 39.7% could be detected in the identified peptides. Thus, in other words, from the PICS results, these recombinant flaviviral proteases show a remarkable promiscuity toward in vitro peptide substrates. Furthermore, another interesting remark can be made: not necessarily the peptide with the most-frequent amino acids in each position (i.e., the canonical sequence) will be found in the peptide pool. The homology model using a WNP-BPTI structure as a template made it possible to identify the S-subsites of the proteases. They show a relatively good degree of conservation between the species, thus structurally partially explaining the conserved amino acid probabilities and promiscuity. As stated by others, the often-found Lysine at P2′ is not compatible with the also often found lysine at P4′, as both substrate subsites partially overlap. Steric interference of this kind could be a possible explanation of why the peptide with the most-probable amino acids in each position often is not found or does not show affinity to specific proteases.

Another interesting feature analyzed here relates to the conservation of promiscuity in *Flavivirus* proteases. As the five tested proteases showed a high extent of common degraded peptides, this indicates that the common flaviviral protease ancestry could be linked to its protease specificity. In order to explain both promiscuity and specificity, a machine learning approach was chosen. This field of computational research has been recently revolutionized by development of the attention-based transformer neural network architecture and the implementation of large language models, like, e.g., ChatGPT.^46^ Recently, these models have been successfully expanded to proteomics research.^47,48^ The rationale behind LLMs is that token sequences can be analyzed by the so-called self-attention mechanism so as to weigh the importance of each token in relation to every other token in a sequence, regardless of their positions. Transposition of this mechanism to proteins would mean that amino acids in a protein sequence could be related to other amino acids in other parts of the protein sequence. By masking randomly amino acids and training the network with huge amounts of protein sequences, the algorithm would—in the computational sense—then be able to create in its neural network weighting scheme an approximate “understanding” of hidden important protein-coded information, which would be reminiscent of deciphering a higher order protein “life code”.^28^ Thus, far, results obtained by pLMs like ProtTrans are very encouraging in that they indeed can be used for predicting of protein sequence-related properties, like its secondary structure prediction and its compartmentalization.^31^

ProtTrans can be used for both regression and classification tasks, and its properties can be described on “per-residue” or “per-protein” basis. Here, the ProtT5-XL UniRef50, which was originally trained with 45 × 10^6^ protein sequences with a total of 14 × 10^9^ amino acids, was used after fine-tuning using the LoRA algorithm with the peptides found in the PICS analysis and random decoys from mouse proteome after adding a classification head in form of a linear neural network to its last transformer layer. As the main result, this model can predict with a test-set accuracy of up to 76.3% if a peptide will be degraded or not by one of the flaviviral proteases. This is an interesting result, as it shows that ProtTrans can be used to predict protein-peptide interactions and potentially biologically relevant substrates. The rationale behind this is that after training, the T5 encoder “learns” information about sequences that are found in one protein that can be applied to estimate probabilities of a peptide binding to protein sequences. To quantify the contribution of the transfer learning step, we compared the LoRA fine-tuned model against a baseline using frozen ProtTrans embeddings. While the pretrained model achieved a reasonable baseline performance (Accuracy: 72.2%, ROC-AUC: 0.81), the fine-tuning process significantly enhanced predictive power, raising the accuracy to 76.3% and the ROC-AUC to 0.88 (Figure S5). This confirms that adapting the model parameters is essential for more accurate predictions of the flaviviral protease specificity.

Both of the output embeddings as the last self-attention matrices of the t5 encoder layers were analyzed for information content. Dimensionality reduction indicated that the fine-tuned T5 output embeddings could indeed contain reminiscent information from their original training and were useful for recognizing protein–peptide interactions. However, the same kind of information on the all-important self-attention matrices from the attention heads could not be detected in the t-SNE analysis (data not shown).

In summary, this work demonstrates an unexpectedly broad substrate promiscuity across proteases from five distinct flaviviral strains, expanding the known specificity profile beyond the classical P1-Arginine motif. Protein language models were successfully deployed to capture this complex specificity. Given the critical role of viral proteases in the replication cycle, the results obtained here offer valuable insights into understanding viral pathogenesis. Furthermore, since many successful antiviral protease inhibitors are based on peptidomimetic scaffolds, these findings could provide a rational basis for implementing novel strategies in antiviral inhibitor design.

## Supplementary Material







## Data Availability

All results here described were obtained by openly available computational methods, as described. All files will be made available as supplementary downloadable content and linked to the final published article.

## Notes

Supplementary methods for principal component analysis and discriminant profiling; sequence logos, PCA biplots, training histories for separated proteases, ROC/t-SNE performance and baseline model comparisons; summary of residue preferences; and detailed training and test metrics for individual protease models (PDF)
Data sets S1–S12 (XLSX)
Peptide target and decoy sequences and data sets S13–S18 providing fine-tuned model weights for the individual and combined protease data sets (ZIP)

## Notes

The mass spectrometry proteomics data have been deposited in the Mass Spectrometry Interactive Virtual Environment (MassIVE, https://massive.ucsd.edu/ProteoSAFe/static/massive.jsp) with the data set identifier MSV000099115.
