# Supplementary material for: Protein Large Language Models Can Predict Flavivirus Protease Target Specificity
Source: ACS Omega. 2026 Apr 1;11(14):22148–57. doi: 10.1021/acsomega.5c13455 (PMC13084463; doi:10.1021/acsomega.5c13455)
Supplement: Supplementary file 1 [file ao5c13455_si_001.pdf]

Protein Large Language Models Can Predict *Flavivirus* Protease Target Specificity.

Rafael Montilla[a,‡], Letícia Dias Lima Jedlicka[b,c,‡], Uilla Barcick[b], Murilo Salardani[b], Alison Felipe Alencar Chaves[d], Gloria Gallo[a], Marcela Guimarães[a], Camila Coelho[a], Larissa Slivka[a], André Zelanis[b,], Martin Würtele[a,*]*

[a]Department of Science and Technology, Biochemistry and Structural Biology Laboratory, Federal University of São Paulo - UNIFESP, São José dos Campos, São Paulo 12231-280, Brazil

[b]Department of Science and Technology, Functional Proteomics Laboratory, Federal University of São Paulo - UNIFESP, São José dos Campos, São Paulo 12231-280, Brazil

[c]Institute of Health and Biological Studies, Federal University of Southern and Southeastern Pará - UNIFESSPA, Marabá, Pará 68507-590, Brazil

[d]Laboratory of Applied Toxinology, Center of Toxins, Immune-Response and Cell Signaling (CeTICS), Instituto Butantan, São Paulo 05503-000, Brazil

This PDF file includes:

- **Figure S1**: Amino Acid Composition of Target and Decoy Datasets.
- **Figure S2**: Comparative Analysis of Functional Diversity in Substrate Specificity.
- **Figure S3:** Training and validation curves (loss and accuracy) for each individual protease model.
- **Figure S4**: Receiver operating characteristic (ROC) curves and t-SNE plots for peptide classification using both all-protease and individual protease models.
- **Figure S5**: Performance Assessment of the Non-Fine-Tuned Baseline Model.
- **Table S1:** Presented most common amino acid residues at each peptide substrate (P5 to P5') position found in the PICS analysis of the flaviviral proteases.
- **Table S2–S6**: Detailed training metrics for the ProtTrans T5 encoder fine-tuning and binary classification head using target and decoy peptides for each flaviviral protease.
- **Dataset S1–S12 (xlsx file)**: Peptide target and decoys sequences identified by PICS for each individual viral protease.
- **Dataset S13–S18 (TXT file)**: Fine-tuned ProtTrans T5 encoder model weights for each individual protease and the combined dataset.

‡R.M. and L.D.L.J. contributed equally to this work.
*To whom correspondence may be addressed. E-mail: martin.wurtele@unifesp.br or andre.zelanis@unifesp.br

Supplementary Methods

Principal Component Analysis (PCA) and Discriminant Profiling. To map the comparative substrate landscape and identify virus-specific specificity features, PCA was performed on the aggregated peptide specificity profiles. A quantitative feature matrix was constructed where each row represented one of the five viral proteases and each column corresponded to the normalized frequency of a specific amino acid at a defined subsite position (P5 to P5'). Prior to dimensionality reduction, invariant features (zero variance) were removed. The data were centered (mean subtraction) but not scaled by standard deviation (Covariance PCA) to ensure that the analysis preserved the biological magnitude of frequency shifts, allowing dominant residues (e.g., P1-Arginine) to appropriately weight the projection. To identify strain-specific sequence preferences, we performed a signed discriminant analysis. For each virus, a discriminant score was calculated for every amino acid at each position as the difference between the frequency in that virus and the mean frequency of the other four strains. This metric distinguishes between specific enrichment (positive score) and specific depletion (negative score) of residues compared to the group consensus.

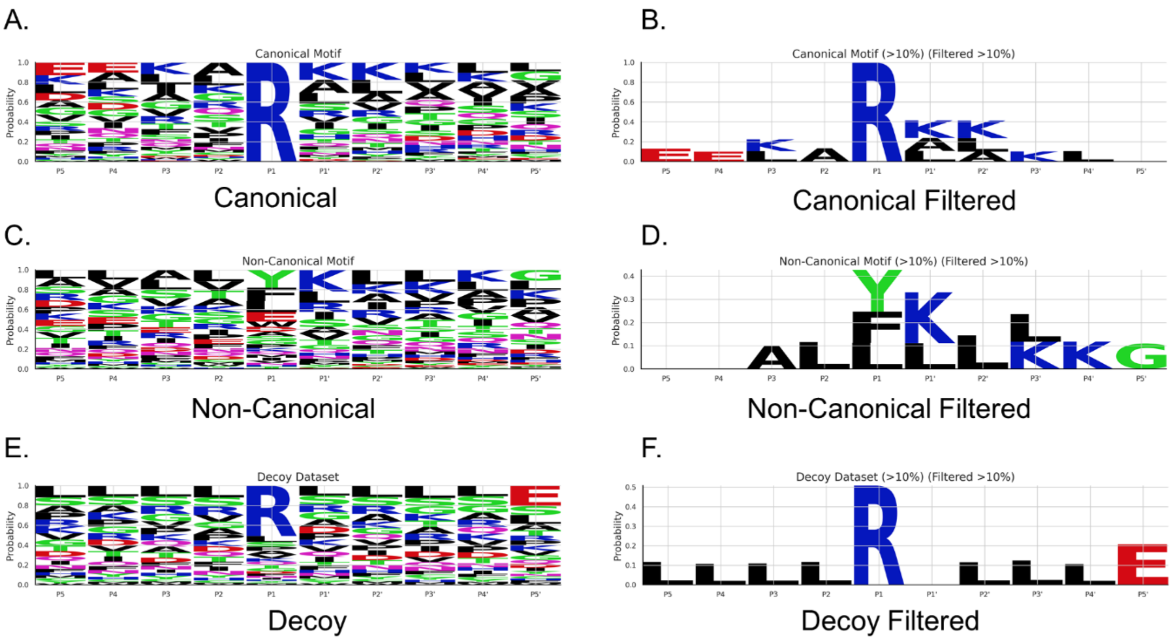

Figure S1. Amino Acid Composition of Target and Decoy Datasets.

Sequence logos visualizing the frequency of amino acids at positions P5 to P5' for the different peptide subsets. **(A, B)** Canonical targets (Arg in P1): Panels show the raw distribution (A) and filtered motifs (>10% frequency) (B), highlighting the dominance of the P1-Arginine. **(C, D)** Non-canonical targets (No P1-Arg): Panels show the raw (C) and filtered (D) motifs, revealing the compensatory enrichment of bulky/hydrophobic (Tyr, Phe, Leu) and Basic (Lys) residues at P1 and P1'. **(E, F)** Decoy Peptides: The decoy set shows the intentional balancing of Arginine at P1 to match the target set, but displays a random distribution of amino acids at all flanking positions, confirming its suitability as a negative control for sequence-context learning.

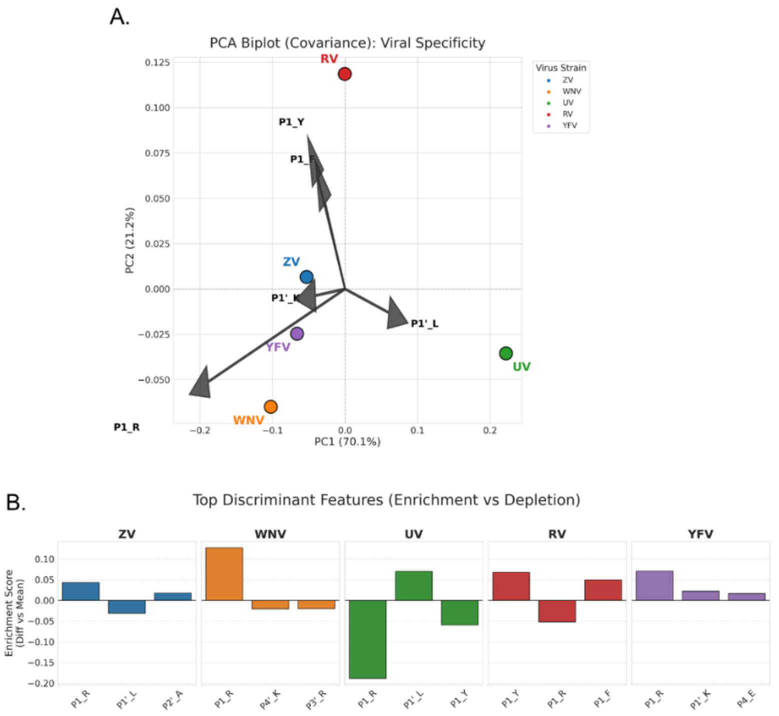

Figure S2. Comparative Analysis of Functional Diversity in Substrate Specificity. (A) Principal Component Analysis (PCA) Biplot. A projection of the average substrate specificity profiles of the five viral proteases onto the first two principal components. The analysis reveals a functional separation of viral strains based on distinct subsite preferences. PC1 (Horizontal) is primarily driven by the P1-Arginine frequency, distinguishing between proteases with strong canonical specificity (West Nile Virus, WNV) and those with divergent profiles (Usutu Virus, USUV). PC2 (Vertical) captures secondary variations in prime-side specificity. Vectors (arrows) represent the PCA loadings (eigenvectors scaled by the square root of eigenvalues), indicating the magnitude and direction of the specific residue-position features driving this functional separation. **(B) Discriminant Analysis.** Bar charts displaying the signed discriminant scores for each virus relative to the group mean. West Nile Virus (WNV) acts as a specificity "specialist," exhibiting a strong positive enrichment for the canonical P1-Arginine, indicating strong adherence to the classical motif. In contrast, Usutu Virus (USUV) displays a significant negative score for P1-Arginine, reflecting a depletion of this residue compared to the group consensus. Rocio Virus (ROCV) is defined by a unique and strong preference for bulky hydrophobic residues (Tyrosine and Phenylalanine) at the P1 position.

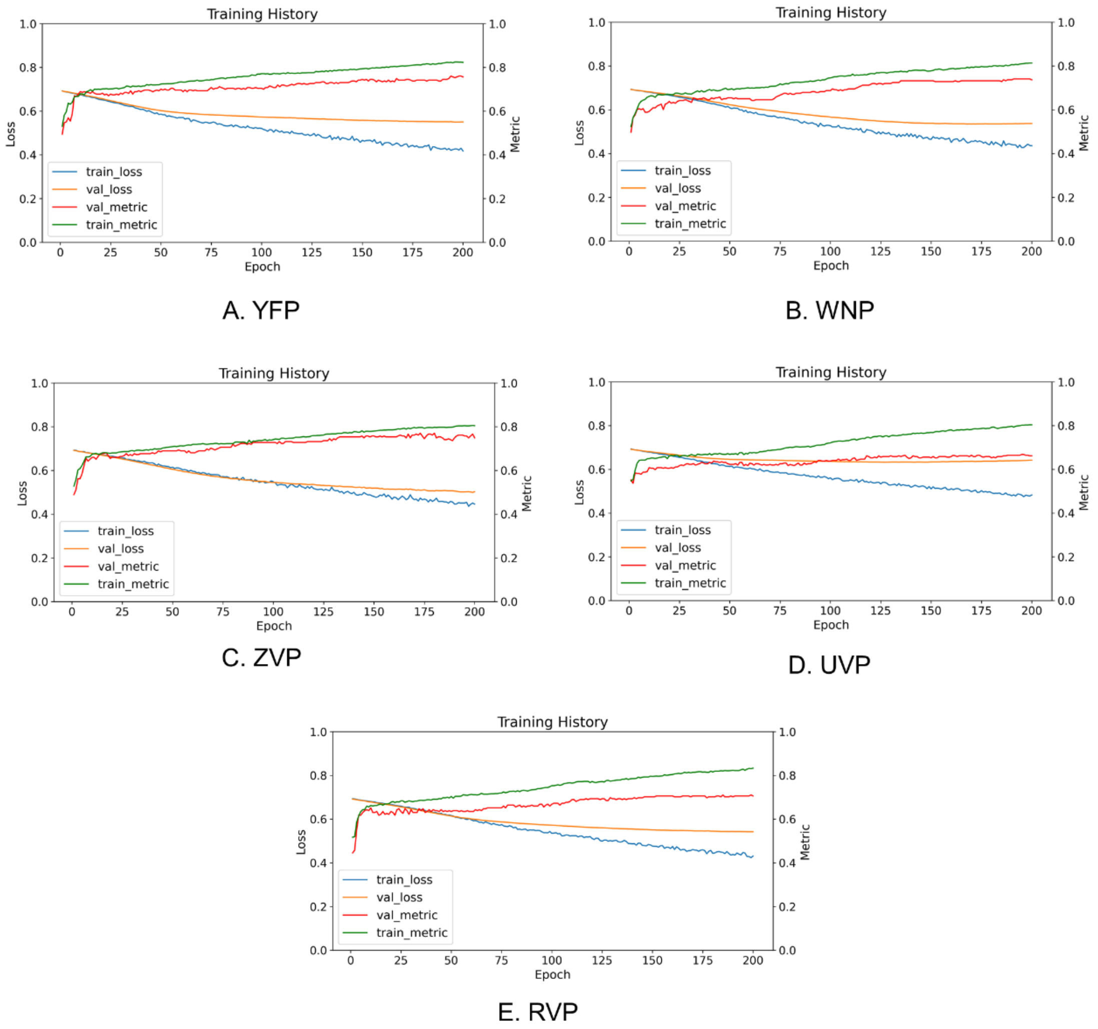

Figure S3. pLM finetuning using target and decoy peptides for separated flavivirus proteases. Training history curves for training and validation sets showing training loss function (train_loss) and validation loss function (val_loss) as well as the training accuracy (train_metric) and validation accuracy (val_metric). A. Yellow Fever virus protease (YFP). B. West Nile Virus Protease (WNP). C. Zika virus protease (ZVP). D. Usutu Virus Protease (UVP). E. Rocio Virus Protease (RVP).

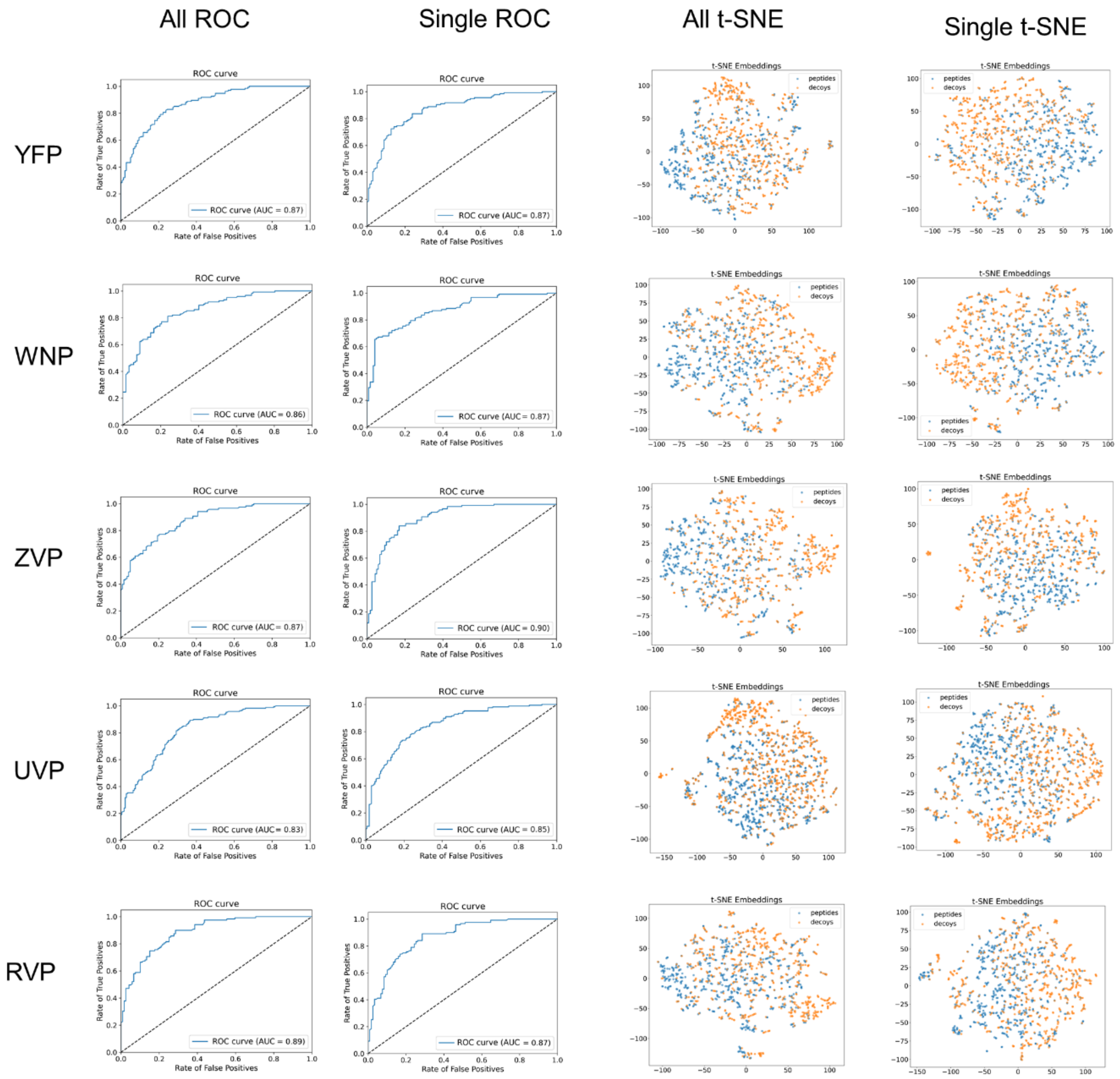

Figure S4. pLM for the single flaviviral proteases. Left. Receiver operating characteristic (ROC) curve of target peptides and decoy peptide classification using the fine-tuned ProtTrans T5 encoder model. All ROC/t-SNE graphs were calculated using the all-protease finetuned model. Single ROC/t-SNE graphs were calculated using the single protease fine-tuned models. **Right.** t-SNE 2D projection representing partial separation of target peptides (blue) and decoy peptide (yellow) embeddings from the fine-tuned ProtTrans T5encoder model.

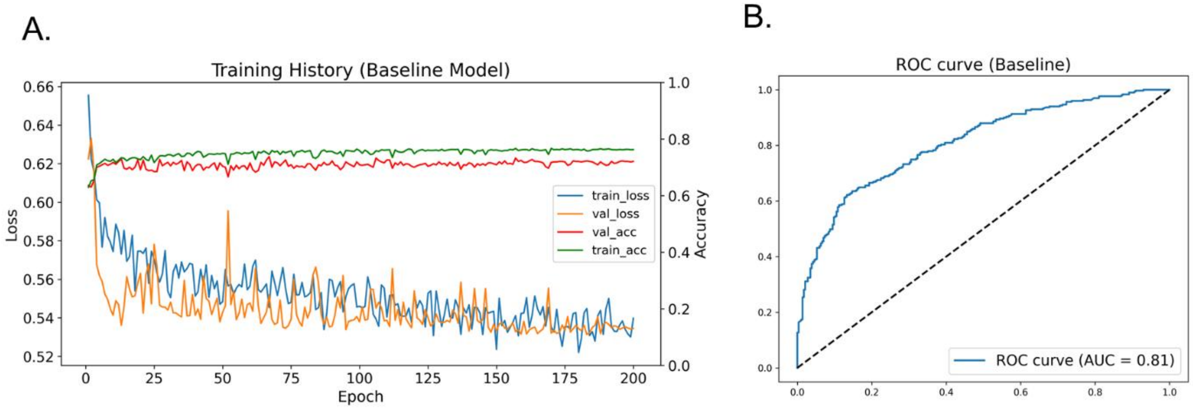

Figure S5. Performance Assessment of the Non-Fine-Tuned Baseline Model. To quantify the specific contribution of the LoRA fine-tuning process, a baseline classifier was trained using frozen ProtTrans-XL embeddings (where the pre-trained Transformer weights were locked, and only the linear classification head was optimized). (A) Training History. The plot displays the evolution of training and validation Loss (Left Axis) alongside training and validation accuracy (Right Axis) over the training epochs. (B) Receiver Operating Characteristic (ROC) Curve. The baseline model achieved an AUC of 0.81 on the independent test set (Test Accuracy: 72.1%). While this baseline performance confirms that the foundation model captures general protein properties, it significantly underperforms compared to the LoRA fine-tuned model (AUC 0.88, Accuracy 76.3%).

Table S1. Most common amino acid residues at each peptide substrate (P5 to P5') position found in the PICS analysis of the flaviviral proteases.

	**P5**	**P4**	**P3**	**P2**	**P1**	**P1'**	**P2'**	**P3'**	**P4'**	**P5'**
**YFP**	E	E	K	A/L	R	K	L/K	L	K	G
**WNP**	E	A	K	A	R	K	L	K	K	L
**ZVP**	E	A	V	L	R	K	L	K	K	P
**UVP**	E	L	L	L	R	K	L	K	K	L
**RVP**	E	A	A	L	R	K	L	K	K	P

YFP: Yellow Fever virus protease. WNP: West Nile Virus Protease. ZVP: Zika virus protease. UVP: Usutu Virus Protease. RVP: Rocio Virus Protease.

Table S2. pLM finetuning with ProtTrans T5 Encoder and binary classification head for the Yellow Fever Virus Protease (YFP) data.

**Train set YFP**					**Test set YFP**				
	Total	*Classified as target*	*Classified as decoy*	*Accuracy [%]*		*Total*	*Classified as target*	*Classified as decoy*	*Accuracy [%]*
canonical targets	281	240	41	85,4	canonical targets	90	65	25	72,2
non-canonical targets	244	209	35	85,7	non-canonical targets	52	43	9	82,7
total targets	525	449	76	85,5	total targets	142	108	34	76,1
canonical decoys	269	59	210	78,1	canonical decoys	64	16	48	75,0
non-canonical decoys	273	53	220	80,6	non-canonical decoys	61	16	45	73,8
total decoys	542	112	430	79,3	total decoys	125	32	93	74,4
	Total	*Correctly classified*	*Wrongly classified*	*Accuracy [%]*		*Total*	*Correctly classified*	*Wrongly classified*	*Accuracy [%]*
total peptides	1067	879	188	82,4	total peptides	267	201	66	75,3

Table S3. pLM finetuning with ProtTrans T5 Encoder and binary classification head for the West Nile Virus Protease (WNP) data.

Train set WNP					Test set WNP				
	Total	*Classified as target*	*Classified as decoy*	*Accuracy [%]*		*Total*	*Classified as target*	*Classified as decoy*	*Accuracy [%]*
canonical targets	288	244	44	84,7	canonical targets	77	62	15	80,5
non-canonical targets	194	167	27	86,1	non-canonical targets	48	34	14	70,8
total targets	482	411	71	85,3	total targets	125	96	29	76,8
canonical decoys	245	61	184	75,1	canonical decoys	58	24	34	58,6
non-canonical decoys	244	43	201	82,4	non-canonical decoys	60	13	47	78,3
total decoys	489	104	385	78,7	total decoys	118	37	81	68,6
	Total	*Correctly classified*	*Wrongly classified*	*Accuracy [%]*		*Total*	*Correctly classified*	*Wrongly classified*	*Accuracy [%]*
total peptides	971	796	175	82,0	total peptides	243	177	66	72,8

Table S4. pLM finetuning with ProtTrans T5 Encoder and binary classification head for the Zika Virus Protease (ZVP) data.

Train set ZVP					Test set ZVP				
	Total	*Classified as target*	*Classified as decoy*	*Accuracy [%]*		*Total*	*Classified as target*	*Classified as decoy*	*Accuracy [%]*
canonical targets	246	192	54	78,0	canonical targets	67	40	27	59,7
non-canonical targets	216	174	42	80,6	non-canonical targets	57	45	12	78,9
total targets	462	366	96	79,2	total targets	124	85	39	68,5
canonical decoys	236	34	202	85,6	canonical decoys	57	11	46	80,7
non-canonical decoys	239	53	186	77,8	non-canonical decoys	54	10	44	81,5
total decoys	475	87	388	81,7	total decoys	111	21	90	81,1
	Total	*Correctly classified*	*Wrongly classified*	*Accuracy [%]*		*Total*	*Correctly classified*	*Wrongly classified*	*Accuracy [%]*
total peptides	937	754	183	80,5	total peptides	235	175	60	74,5

Table S5. pLM finetuning with ProtTrans T5 Encoder and binary classification head for the Usutu Virus Protease (UVP) data.

Train set UVP					Test set UVP				
	Total	*Classified as target*	*Classified as decoy*	*Accuracy [%]*		*Total*	*Classified as target*	*Classified as decoy*	*Accuracy [%]*
canonical targets	242	179	63	74,0	canonical targets	49	31	18	63,3
non-canonical targets	422	345	77	81,8	non-canonical targets	121	79	42	65,3
total targets	664	524	140	78,9	total targets	170	110	60	64,7
canonical decoys	341	48	293	85,9	canonical decoys	75	18	57	76,0
non-canonical decoys	328	73	255	77,7	non-canonical decoys	89	34	55	61,8
total decoys	669	121	548	81,9	total decoys	164	52	112	68,3
	Total	*Correctly classified*	*Wrongly classified*	*Accuracy [%]*		*Total*	*Correctly classified*	*Wrongly classified*	*Accuracy [%]*
total peptides	1333	1072	261	80,4	total peptides	334	222	112	66,5

Table S6. pLM finetuning with ProtTrans T5 Encoder and binary classification head for the Rocio Virus Protease (RVP) data.

Train set RVP					Test set RVP				
	Total	*Classified as target*	*Classified as decoy*	*Accuracy [%]*		*Total*	*Classified as target*	*Classified as decoy*	*Accuracy [%]*
canonical targets	228	184	44	80,7	canonical targets	44	30	14	68,2
non-canonical targets	246	205	41	83,3	non-canonical targets	76	54	22	71,1
total targets	474	389	85	82,1	total targets	120	84	36	70,0
canonical decoys	240	35	205	85,4	canonical decoys	57	14	43	75,4
non-canonical decoys	236	40	196	83,1	non-canonical decoys	61	20	41	67,2
total decoys	476	75	401	84,2	total decoys	118	34	84	71,2
	Total	*Correctly classified*	*Wrongly classified*	*Accuracy [%]*		*Total*	*Correctly classified*	*Wrongly classified*	*Accuracy [%]*
total peptides	950	790	160	83,2	total peptides	238	168	70	70,6
